# The Ovary–Brain Connection

**DOI:** 10.3390/cells13010094

**Published:** 2024-01-02

**Authors:** Abdelrahman Yousif, Ahmed Ebeid, Balint Kacsoh, Martina Bazzaro, Ilana Chefetz

**Affiliations:** 1 Department of Obstetrics and Gynecology, Texas Tech University Health Sciences Center, El Paso, TX 79905, USA; 2 Department of Obstetrics and Gynecology, The George Washington University School of Medicine and Health Sciences, Washington, DC 20037, USA; 3 Department of Biomedical Sciences, Mercer University School of Medicine, Macon, GA 31207, USA; 4 Masonic Cancer Center and Department of Obstetrics, Gynecology and Women’s Health, University of Minnesota, Minneapolis, MN 55455, USA; 5 Department of Biomedical and Clinical Science, Linköping University, SE-581 85 Linköping, Sweden

## 1. Introduction

The brain and the ovaries are in a state of continuous communication. This ovarian–brain connection is essential for regulating physiological functions. Any alteration can lead to pathological conditions in both organs, e.g., central amenorrhea or altered cognitive functions, mood, and behaviors [1,2,3,4]. Ovarian hormones (mainly estrogen and progesterone) can cross the blood–brain barrier and exert their physiological functions on cortical and subcortical targets. Conversely, the hypothalamus and the pituitary gland also secrete gonadotropin-releasing hormone and gonadotropins, respectively, that affect the ovarian secretion of steroid hormones, also called the hypothalamic–pituitary–ovarian (HPO) axis [5]. For example, the fluctuations in ovarian hormone levels are responsible for normal menstrual cycle-related mood and behavior changes and contribute to the pathophysiology of medical conditions like migraine headaches [6]. Women of postmenopausal age are at increased risk of age-related dementia more than their male counterparts, suggesting a role of the menopausal decline in sex steroids in this finding [7]. Similar observations were made in a Mayo Clinic cohort study of oophorectomy and aging that demonstrated an increased risk of cognitive impairment in patients who had undergone bilateral oophorectomy (surgical menopause) compared to women who did not undergo oophorectomy [8]. In this article, we highlight some common pathways with important activities in the ovaries and the brain in benign and malignant conditions and the consequences of their disruption in disease development by reviewing articles published in the current Special Issue.

## 2. Proposed Shared Pathways for Disease Development in the Ovary and the Brain

### 2.1. Histone Modifications

One of the shared abnormalities between ovarian and brain tumors is the modification of histones, a process that can change how genes are expressed [9]. One of the studies on this issue conducted by Day et al. focused on a specific type of histone modification known as the post-translational modification of the core histones. The study examined H3K27, a member of the core histone H3 family that undergoes methylation [10,11]. This process is carried out by a protein complex known as Polycomb Repressive Complex 2 (PRC2), which includes an enzyme called Enhancer of Zeste Homolog 2 (EZH2) [12,13]. It is of interest to note that the methylated form of H3K27 is subject to certain dysregulations that contribute to the development of two vastly distinct cancer types: diffuse midline gliomas (DMGs) and various forms of ovarian cancer, including malignant epithelial tumors such as serous cystadenocarcinomas, mucinous cystadenocarcinomas, endometrioid carcinomas, clear cell tumors, and non-epithelial ovarian cancers [14]. DMGs are common high-grade tumors in pediatric patients that occur in midline neural structures such as the spinal cord, thalamus, and brainstem [15]. Studies have revealed that the loss of H3K27 methylation is a crucial driver of tumorigenesis in DMGs, as it has been observed that the H3 K27M mutation inhibits the auto methylation of EZH2 by PRC2 [16]. In the same context, a promising approach has been developed for treating K27M-expressing brain stem gliomas by inhibiting the K27 demethylase enzyme, thereby restoring the cellular H3K27 methylation in these tumor cells. A study conducted in 2014 demonstrated a potent antitumor activity via the inhibition of the K27 demethylase in K27M mutant glioma cells. This antitumor activity was noticed not only in vitro in K27M cells but also in vivo when these tumor cells were implanted into the pontine tegmentum of mice as xenografts [17]. Conversely, in epithelial ovarian cancer (EOC), hypermethylation of H3K27, caused by the overexpression of EZH2, has been shown to not only correlate with high tumor vascularization but also the high tendency of tumor metastasis leading to worse outcomes [14,18,19,20,21]. In a study conducted by Guo et al. to determine the role of EZH2 and p53 in ovarian cancer, EZH2 was abundantly present in more than 80% of EOCs but only 37.5% of non-epithelial ovarian cases [22].

### 2.2. Glucose Metabolism

Under anaerobic conditions, normal tissues generate adenosine triphosphate (ATP) by fermentation, i.e., utilizing only the glycolytic pathway, which produces lactate from glucose. When the same tissue is exposed to an aerobic environment, biochemical feedback mechanisms greatly decrease glycolysis. The tricarboxylic acid (TCA) cycle and oxidative phosphorylation achieve markedly increased ATP generation. In 1930, Otto Warburg termed the decrease in glycolysis in response to aerobic conditions the Pasteur effect. Rapidly dividing cancer cells preferentially use a glycolytic anaerobic pathway even in the presence of oxygen, known as the Warburg effect [23]. Adenosine monophosphate (AMP)-activated protein kinase (AMPK) acts as a sensor for the cellular adenosine diphosphate (ADP) and AMP levels. When cellular energy levels are low (the AMP/ATP ratio is high), as is often the case in cancer cells due to their rapid growth and altered metabolism, AMPK (a heterotrimer complex that exists in various isoforms) becomes activated. AMPK activation in cancer cells stimulates glycolysis. AMPKs play an essential role in tumor progression. Liver Kinase B1 (LKB1) is a key activator for AMPK. There are several downstream targets for the LKB1-AMPK pathway, including but not limited to Novel (nua) kinase family 1 (NUAK1) and NUAK2 [24]. According to The Cancer Genome Atlas (TCGA) program, dysregulation of NUAK1 and/or NUAK2 was found in several cancers, e.g., ovarian, breast, lung, and liver cancers. Downregulation of NUAK1 was linked to reduced invasiveness in renal and breast cancer cell lines [25]; meanwhile, its overexpression was associated with poor prognosis in the murine model of colorectal cancer [26]. Different mutations of NUAK have been described in the literature. NUAK1 lies on the short arm of chromosome 12, while NUAK 2 is on the short arm of chromosome 1. NUAK1 is expressed mainly in the brain, skin, and to a lesser extent in the breast, ovary, heart, and lung tissues; meanwhile, NUAK2 is found in the kidneys, ovary, breast, small bowel, and colon [27]. Molina et al. reported that Cbioportal data showed genetic NUAK1 and NUAK2 alterations in ovarian and breast cancers. Furthermore, the same paper demonstrated that up-regulated NUAK1 was associated with poor survival after assessing glioma patient samples (*p* < 0.005). Similar results were found using the Protein Atlas database, indicating poor survival in ovarian cancer with NUAK1 overexpression, indicating an association between NUAK1 overexpression and poor survival both in glioma and ovarian cancer [28,29]. NUAK proteins play an important role in tumor growth and invasiveness; here, we highlight the metabolism-associated signaling cascades in which these proteins play a role in both ovarian and brain cancers.

## 3. Microtubule-Associated-Proteins (MAPs) and Microtubule Stability

Regulation of microtubule (MT) stability is crucial to essential cellular functions such as growth, differentiation, and intracellular transport [30]. Microtubule-associated proteins (MAPs) are a diverse group of proteins that interact with MTs, regulating their dynamic instability, and they can be broadly categorized into two groups: MT-stabilizing and MT-destabilizing (including MT-severing) proteins [31,32]. Some well-known MAPs include Tau, MAP2, and MAP4, primarily associated with neurons, as well as MAP1, MAP6, and stathmins found in various cell types. Stathmin-2, for instance, is an MT-destabilizing MAP that regulates MT dynamics by sequestering tubulin dimers, thus slowing down MT growth from their plus-end; its premature loss has been associated with neurodegeneration in amyotrophic lateral sclerosis and frontotemporal dementia [33]. In ovarian cancer, overexpression of stathmin is associated with poor prognosis and resistance to MT-targeting chemotherapeutic agents like paclitaxel [34]. On the other hand, Tau is an MT-stabilizing MAP and a well-characterized key contributor to a class of neurogenerative diseases called tauopathies [35,36,37]. Recent studies have revealed Tau’s presence in ovarian cancer cells. Its aberrant expression was associated with ovarian cancer cells’ migration and invasion and suggested to be a contributor to tumor progression [38,39,40].

A study by Clemente et al. in this Special Issue found that UNC-45A, a cytoskeletal protein with dual and non-mutually exclusive role as a myosin II chaperone and microtubule-severing protein, is enriched in proliferating cells in the mouse upper genital tract, specifically in the mature follicles during proestrus, but is also present in the oocyte and the ciliated epithelium of the fallopian tube [41,42,43,44]. These regulators are important for ovarian cancer cell proliferation (related to the mitotic spindle) and neuronal development (related to microtubular transport mechanisms). The expression of UNC-45A is also observed in neurons in the mouse nervous system, particularly in areas containing axons and dendrites [45]. It is also overexpressed in breast and ovarian cancer and, even more so in ovarian cancers resistant to the microtubule-stabilizing drug paclitaxel [46]. More recently, the same group demonstrated that UNC-45A preferentially binds to curved microtubules and that in the presence of UNC-45A, cellular microtubules increase their curvature and break [47]. Notably, UNC-45A is the only MT-severing protein we know of that does not have an ATPase domain, and several human diseases (such as cancer and neurodegenerative diseases) are characterized by both a decrease in ATP levels and a highly oxidative environment [48,49]. In this scenario, UNC-45A may have a functional advantage over other MT-destabilizing proteins and a significant role in human diseases from cancer to neurodegeneration. Lastly, others have found that the loss-of-function mutations in UNC-45A led to a syndrome characterized by symptoms such as diarrhea, cholestasis, bone fragility, impaired hearing, and intellectual disability [50].

### 3.1. Tumor Innervation

In a study reported in this issue [51], Barr et al. investigated the role of tumor innervation in ovarian cancer using a syngeneic model that mimics human ovarian cancers. They found that sensory nerves innervate high-grade serous ovarian cancer (HGSOC) tumors. The source of these tumor-infiltrating nerves was traced to the dorsal root ganglia and the inferior vagal (nodose) ganglia, and it was found that the nerves sprout from existing local peripheral nerves. The authors hypothesize that the tumor microenvironment mimics an injury environment, which signals the axons to sprout and innervate the tumor [52]. The study also found that the majority of the nerves in the tumor are near blood vessels and that tumor-induced nerve sprouting occurs at these sites. Pain is a common problem among patients with advanced ovarian cancer, and it is suggested that sensory innervation arising from tumor-induced nerve sprouting may contribute to the microenvironment that supports tumor growth.

### 3.2. Purine Biosynthesis and Metabolism

Purine biosynthesis, a process comprised of 11 enzyme-catalyzed reactions, is highly dependent on the TCA cycle and its associated metabolites. Nucleotide biosynthesis and its mitochondrion-dependent pathways are crucial for cancer cell survival [53]. In particular, glutamine -which, via glutamate, is a precursor of α-ketoglutarate, one of the components of the TCA cycle- is highly utilized for purine biosynthesis by rapidly dividing cancer cells through the upregulation of glutamine synthetase enzyme, which competes for glutamate’s usage in the TCA cycle [54]. This metabolic process has been reported in ovarian cancer, where highly invasive cells are markedly glutamine-dependent [55]. It is well known that there are two distinct pathways for purine biosynthesis: the de novo pathway—which demands high-energy metabolic needs- and the salvage pathway. Both lead to the formation of ATP and GTP as end-products. In a study [56] using mice as hosts for patient-derived glioblastoma xenografts, Kofuji et al. concluded that glioblastoma tissues consume GTP, preferentially produced via the de novo pathway. Furthermore, they found a two-fold increase in the expression of the gene that encodes IMP dehydrogenase 2 (IMPDH2), the rate-limiting cytosolic enzyme of de novo guanine nucleotide biosynthesis, in ten genetically different mouse gliomas vs control brains (*p* < 0.05).

### 3.3. Ovarian Cancer Metastasis to the Brain

Brain metastases (BMs) in ovarian cancer patients are relatively uncommon, between 0.3% and 4.6% [57,58], but are associated with poor survival. Serous EOC is the most common histologic subtype with BM in the available literature [59]. Comorbidities associated with BMs in EOC patients largely depend on the location of the lesions but also potentially manifest as neurocognitive decline. To date, the mainstay treatments for BMs are either surgery or radiotherapy. A study by Limon et al. [60] showed that the risk of BM arising from EOC with Breast Cancer gene (BRCA) mutations is increased compared to the risk of BM arising from EOC with wild-type BRCA. The study did not demonstrate statistical significance in overall survival between the two groups. In this editorial, we draw attention to a comprehensive review by Scotto et al. [61] featured in the current issue, reviewing the latest literature on biomarkers for the development of BMs in women with EOC.

### 3.4. Biomarkers for Ovarian Metastasis to the Brain

Hormonal receptors have been studied as markers of the potential spread of EOC to the central nervous system. For example, estrogen receptor- (ER-) α overexpression was found in ovarian cancer tissue, enhancing the epithelial-mesenchymal transition and development of metastasis [62,63]. While ER-α isoform has been known to support tumor growth, ER-β has been suggested to function as a tumor suppressor. Indeed, experimental results showed that only ER-α is expressed in metastatic cells [62]. A literature review suggested that ER-β localization and shuffling between cytosol and mitochondrion plays an important role in drug response [64]. Hormonal receptors may play a role as therapeutic targets. However, to date, no studies have been reported on hormonal therapy for EOC patients with BM [61]. Other markers, such as BRCA and multidrug resistance protein (MDR)-1, have been studied [58,65,66]. For example, a study on 340 cases of EOC showed only 7 patients with BM, 4 of whom had a loss of heterozygosity and two who had germline mutations of BRCA [65]. Ratner et al. [58,60] found a higher prevalence of BMs in EOC patients with BRCA mutation compared to wild-type BRCA counterparts. MDR-1 and its association with metastasis development have been studied in EOC patients. Matsu et al. [66] showed higher expression of MDR-1 in patients with BMs compared to patients with relapses in extracranial sites (abdomen, pelvis, spleen, and liver). Exploring different biomarkers in EOC with a higher risk of BM development might assist with the identification of at-risk patients and tailor their follow-up plans accordingly.

### 3.5. The Role of Ovarian Estrogens in Migraine Attacks

Migraine headache is considered one of the most common medical conditions that affect females under the age of 50 years. In this issue, a review by Nappi et al. extensively illustrates the role of estrogens in the pathophysiology of migraines and the available data (of which some are experimental) on the use of estrogens and progestins in the prevention of migraines. Migraines are more common in females than males and are tightly associated with certain reproductive events. Migraine frequency increases around puberty, the premenstrual phase of the menstrual cycle, and declines after menopause [67,68]. Estrogen receptors are found in brain areas that mediate pain perception and sensitivity pathways, e.g., the cerebral cortex, hypothalamus, cerebellum, and limbic system [69,70,71,72]. Reproductive hormones also influence calcitonin gene-related peptide (CGRP), one of the neuropeptides that mediates peripheral and central pain sensory pathways and functions as a vasodilator via an axonal reflex [73]. Several studies found higher levels of GCRP in females compared to males, during pregnancy compared to non-pregnant females, and with combined hormonal contraceptive pills use. With the recent literature elucidating the role of GCRP in migraines and its use as a possible therapeutic target for migraines headache/pain control, some data showed conflicting results regarding the effect of estrogen on GCRP levels. Interestingly, Wyon et al. [74] found higher levels of GCRP in postmenopausal women (a low estrogenic condition).
